# Outcome of COVID-19 patients with haematological malignancies after the introduction of vaccination and monoclonal antibodies: results from the HM-COV 2.0 study

**DOI:** 10.1007/s10238-023-01027-y

**Published:** 2023-03-03

**Authors:** Alessandra Oliva, Francesco Cogliati Dezza, Flavia Petrucci, Francesco Eugenio Romani, Matteo Morviducci, Flavio Marco Mirabelli, Francesca Cancelli, Emanuele Valeriani, Giulia Marcelli, Francesco Pugliese, Ombretta Turriziani, Paolo Ricci, Mario Venditti, Paolo Palange, Claudio Maria Mastroianni

**Affiliations:** 1 Department of Public Health and Infectious Diseases, Sapienza University of Rome, Piazzale Aldo Moro 5, 00185 Rome, Italy; 2 Unit of Emergency Radiology, Department of Radiological, Oncological and Pathological Sciences, Sapienza University of Rome, Rome, Italy; 3 Department of Anesthesia and Critical Care Medicine, Sapienza University of Rome, Policlinico Umberto I, Rome, Italy; 4 Microbiology and Virology Laboratory, Department of Molecular Medicine, Sapienza University of Rome, Rome, Italy

**Keywords:** COVID-19, Haematological malignancies, Outcome, Vaccines, Monoclonal antibodies

## Abstract

Patients with haematological malignancies (HM) and SARS-CoV-2 infection present a higher risk of severe COVID-19 and mortality. The aim of the study was to investigate whether vaccination and monoclonal antibodies (mAbs) have modified the outcomes of HM patients with COVID-19. This is a single-centre retrospective study in HM patients hospitalized due to SARS-CoV-2 infection from March 2020 to April 2022. Patients were divided into PRE-V-mAb group (patients hospitalized before the introduction of vaccination and mAbs) and POST-V-mAb group (patients hospitalized after the use of vaccine and mAbs). A total of 126 patients were included (65 PRE-V-mAb and 61 POST-V-mAb). POST-V-mAb patients showed a significantly lower risk of intensive care unit (ICU) admission (8.2% vs. 27.7%, *p* = 0.005), shorter viral shedding [17 (IQR 10–28) vs. 24 days (IQR 15–50), *p* = 0.011] and shorter hospitalization length [13 (IQR 7–23) vs. 20 (IQR 14–41) days, *p* = 0.0003] compared to the PRE-V-mAb group. Nevertheless, both in-hospital and 30-day mortality rates did not significantly differ between the two groups (29.5% POST-V-mAb vs. 36.9% PRE-V-mAb and 21.3% POST-V-mAb vs. 29.2% PRE-V-mAb, respectively). At the multivariable analysis, an active malignancy (*p* = 0.042), a critical COVID-19 at admission (*p* = 0.025) and the need for high-level of oxygen support at respiratory worsening [either HFNC/CPAP (*p* = 0.022) or mechanical ventilation (*p* = 0.011)] were independently associated with in-hospital mortality. In the subgroup of POST-V-mAb patients, receiving therapy with mAbs was a protective factor (*p* = 0.033). Despite the new therapeutic and preventive strategies available, HM patients with COVID-19 disease represent an extremely vulnerable group with still high mortality rates.

**Supplementary Information:**

The online version contains supplementary material available at 10.1007/s10238-023-01027-y.

## Introduction

SARS-CoV-2 is the etiological agent of COVID-19, and since December 2019, it has rapidly spread around the world causing the most significant global pandemic in the last century [1]. Nowadays, the international knowledge about COVID-19 continues to evolve, and recently, data suggest that severe and critical disease can occur in up to 15 and 5% of patients, respectively [2].

However, immunosuppression could lead to more severe disease and mortality [3], with patients suffering from haematological malignancies (HM) presenting the highest risk of adverse outcomes compared to the general population [4–7].

Indeed, HM patients have an increased chance to be admitted to intensive care units (ICUs), a prolonged viral shedding with longer hospitalization time and a higher mortality rates than subjects without HM [4, 6, 8–12], especially in the presence of acute myeloid leukaemia and myelodysplastic syndromes [4, 13].

The vulnerability of this population is explained by the severe immunosuppression resulting from both chemo-immunotherapy and the underlying disease [3]; in addition, the long-lasting persistence of the virus may contribute to worse outcome and selection of new variants.

Since the first pandemic wave, the scenario has radically changed due to the widespread of SARS-CoV-2 vaccination and the availability of early therapies including antiviral drugs and monoclonal antibodies (mAbs) [14]. However, data have shown a low rate of seroconversion in patients with HM fully vaccinated with two doses of anti-SARS-CoV-2 mRNA vaccine, especially if previously treated with anti-CD20 antibodies [15]. Moreover, a third vaccine does not induce seroconversion in patients who have not responded before even if it cannot be ruled out the presence of possible protective cellular T cell response [16].

Monoclonal antibodies (mAb) such as sotrovimab, casirivimab–imdevimab and bamlanivimab–etesevimab or the recently introduced long-acting one have provided valuable options for the treatment of COVID-19 disease; this may be especially true for patients with immunocompromised conditions [17, 18], who may also benefit from convalescent plasma [19, 20].

However, whether these new available strategies have changed the outcomes in HM patients with SARS-CoV-2 is still under investigation.

Based on these considerations, we aimed to evaluate the clinical characteristics and outcomes (in-hospital mortality, ICU admission and duration of viral shedding) in hospitalized patients with HM and COVID-19 after the introduction of vaccination and mAb. Furthermore, we evaluated the risk factors for prolonged viral shedding in this population.

## Materials and methods

From March 2020 to April 2022, a retrospective, single-centre study was performed in a cohort of patients with HM and COVID-19 hospitalized at an Academic Hospital in Rome. Inclusion criteria were: (i) diagnosis of COVID-19 by means of molecular/antigen tests, (ii) hospitalization and (iii) age > 18 years. Patients with haematological diseases other than malignancy and age < 18 years were excluded from the study. The cohort of patients was further divided into two groups: the PRE-V-mAb group (patients hospitalized before the introduction of vaccination, mAbs and oral antiviral) and the POST-V-mAb group (patients hospitalized after the use of vaccine, mAbs and oral antiviral).

**Fig. 1:**
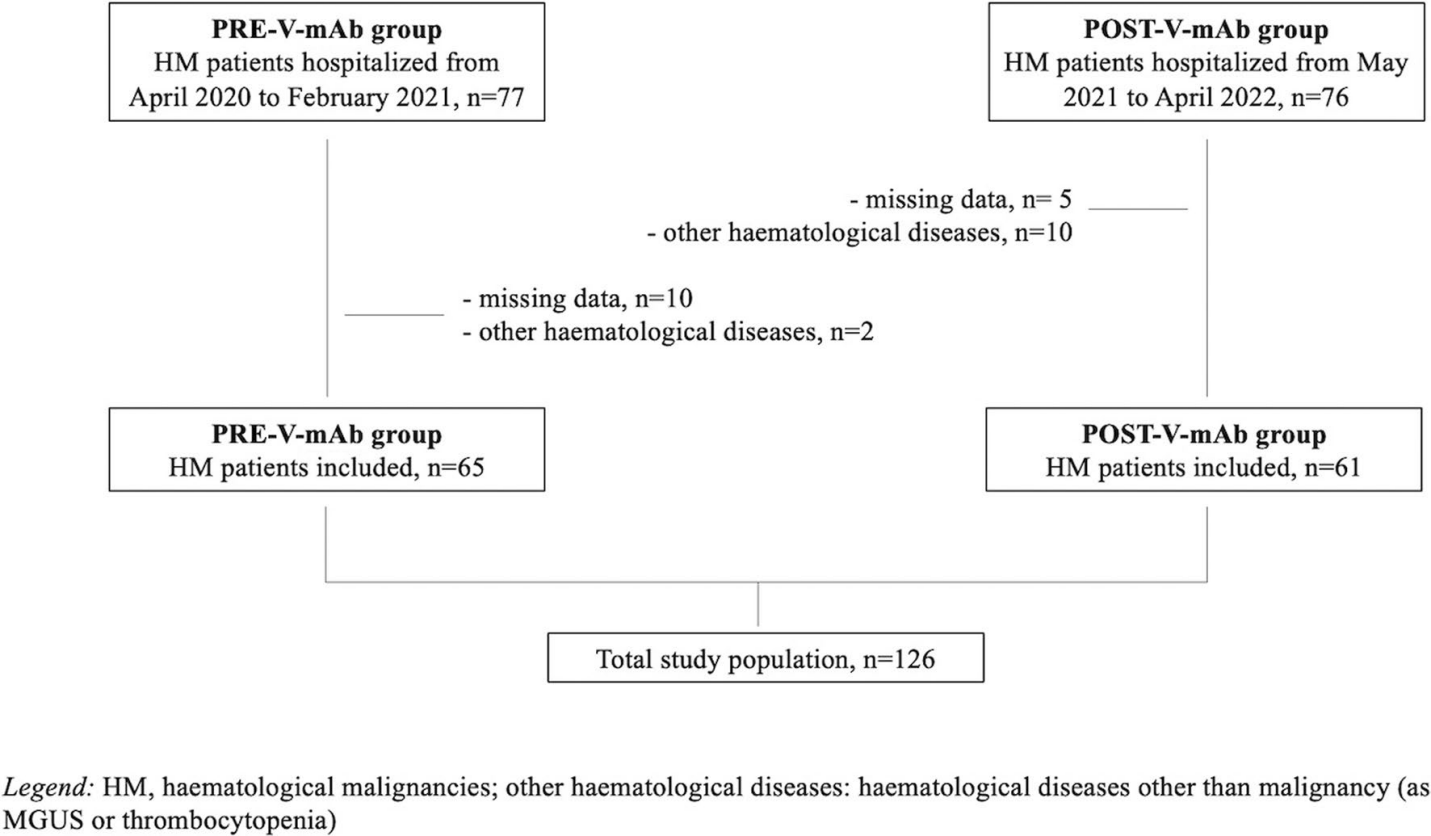
Flow chart of the study population. HM: haematological malignancies

At our institution, vaccination to patients with HM started in January 2021, whereas mAb and oral antivirals were available from May 2021 and January 2022, respectively. Therefore, the PRE-V-mAb group included patients enrolled from March 2020 to February 2021 and the POST-V-mAb group from May 2021 to April 2022 (Fig. 1). Accordingly, we had only 3 months to include patients treated with oral antivirals.

Nasopharyngeal swab samples were collected, and SARS-CoV-2 RNA was detected using real-time RT-PCR assay (RealStar SARS-CoV2 RT-PCR, Altona Diagnostics). For each patient, laboratory and clinical data at hospital admission and during hospitalization were collected and recorded anonymously in an electronic database. Therapeutic regimens including mAbs, antiviral therapy and the use of steroids were based on the international and local guidelines available at that time [14, 21] and on clinical judgment, also following consultation with haematologists, when appropriate. According to the available National Drug Agency (AIFA) indication [22], mAbs was administered within 7 days from the first SARS-CoV-2 RNA-positive nasopharyngeal swab. Apart from guidelines indication, the use of monoclonal antibodies (including casirivimab/imdevimab, bamlanivimab/etesevimab or sotrovimab), short-course remdesivir or oral antiviral as first-choice treatment in outpatients setting was also based on the hospital pharmacy availability and dominant circulating variants of concern at the time. In this regard, the first Italian Omicron case was detected on 27 November 2021, and the variant became dominant in Italy (99% of cases) at the end of January 2022. So, at the time patients in the POST-V-mAb group were infected, and the most widely circulating variants in Italy were firstly Delta and, for a lesser extent, Omicron [23].

### Definitions

Severity of infection was defined according to the WHO classification available at the time of enrolment [24]. The use of corticosteroids within the previous 30 days included therapy with prednisone or its equivalent at a dose > 0.5 mg/kg/day for at least 1 month. Prior infection and antibiotic therapy were defined as a diagnosis of infection and/or the receival of antibiotics in the 30 days prior to hospital admission, respectively. Status of haematological malignancy was defined as new diagnosis, remission, refractory/relapsing disease or yet to define, according to the guidelines of European Society for Medical Oncology [25]. Active malignancy was defined as patients with new diagnosis or refractory/relapsing disease [25]. Prior active treatment included the receival of chemotherapy or immunotherapy, or both, in the previous 90 days. Immunotherapy included the receival of monoclonal antibodies (rituximab, daratumumab and obinutuzumab) and tyrosine kinase inhibitors (imatinib, ibrutinib, ruxolitinib and venetoclax). Worsening of respiratory conditions was based on the change of PaO_2_/FiO_2_ and was defined as: (i) need of supplementary oxygen therapy or (ii) need of increasing oxygen therapy supplementation in a patient with SARS-CoV2 infection for reasons directly related to the infection, as it was already reported [8].

Time of viral shedding was defined as the number of days from the first viral detection by RT-PCR on nasopharyngeal specimen until the first negative result.

The study was approved by the local ethics committee (ID Prot. 109/2020).

### Radiology

Two multidetector CT scanners (Siemens Somatom go.Now 32 and Somatom Sensation 64; Siemens Healthineers) were used. COVID-19 pneumonia was confirmed by means of the following CT parameters: ground-glass opacity, crazy-paving pattern and pulmonary consolidation. A semi-quantitative CT severity score to identify the percentage of lung parenchyma involved by the infective process was therefore calculated, and then, three main groups were obtained: minimum involvement (0–30%), medium involvement (31–60%) and high involvement (> 60%) [26].

### Statistical analyses

Continuous data were expressed as median (interquartile range, IQR), and categorical data were summarized as number of observations (*n*) and percentages (%). Univariable analysis was used to identify risk factors and predictors for all-cause in-hospital mortality. Baseline predictors possibly associated with the outcome at univariable comparison and variables considered clinically significant were considered for multivariable Cox regression analysis. Survival was analysed by Kaplan–Meier curves, and statistical significance of the differences between the groups was assessed using the log-rank test. A logistic multivariable model was constructed to evaluate the predictors of prolonged viral shedding (PVS), which has been considered > 20 days according to the median value of viral shedding in our study population (20.5 days). All statistical analyses were performed with SATA/IC software (StataCorp) version 17.

## Results

### General characteristics

Overall, 126 patients were included in the study, of which 65 PRE-V-mAb group and 61 POST-V-mAb group, respectively. General characteristics of the population are shown in Table 1. Gender distribution as well as patients’ comorbidities, symptoms and disease severity at the admission did not differ significantly between the two groups, while patients in the POST-V-mAb were older [71 (58–80) vs. 64 (50–74) years, *p* = 0.012]. Notably, a similar percentage of patients in both groups had pneumonia at hospital admission (84.6% vs. 78.6%, *p* = 0.389) and developed a respiratory worsening during hospitalization (58.5 vs. 44.3%, *p* = 0.111).

**Table 1:** General features of study population

	Total (*n* = 126)	PRE-V-mAb (*N* = 65)	POST-V-mAb (*N* = 61)	*p* value
Demographics				
Gender (Female), *n* (%)	65 (51.5)	32 (49.2)	33 (50.7)	0.597
Age, median (IQR)	66 (52–77)	64 (50–74)	71 (58–80)	**0.012**
Steroids in the last 30 days before admission, *n* (%)	43 (34.4)	25 (38.5)	18 (29.5)	0.320
Infection in the last 30 days before admission, *n* (%)	23 (18.3)	13 (20)	10 (16.4)	0.600
Antibiotic therapy in the last 30 days (excluded prophylaxis), *n* (%)	39 (31.0)	21 (32.3)	18 (29.5)	0.734
Antibiotic prophylaxis therapy, *n*. (%)	42 (33.3)	23 (35.4)	19 (31.1)	0.614
Days of hospitalization, median (IQR)	16 (9–38)	20 (14–44)	13 (7–23)	**0.0003**
Days from symptoms onset to hospitalization, median (IQR)	3 (1–7)	3 (1–7)	3 (1–7)	0.769
Comorbidities, *n* (%)				
Hypertension	52 (41.3)	22 (33.8)	30 (49.2)	0.081
Coronary artery disease	28 (22.2)	14 (21.5)	14 (22.9)	0.849
Diabetes	17 (13.5)	8 (12.3)	9 (14.8)	0.688
Chronic obstructive pulmonary disease	8 (6.3)	4 (6.1)	4 (6.56)	0.926
Chronic renal failure	9 (7.1)	6 (9.2)	3 (4.9)	0.348
Disease severity at the admission, *n* (%)				
Moderate	80 (63.5)	41 (63.08)	39 (63.93)	0.920
Severe	31 (24.6)	18 (27.7)	13 (21.3)	0.406
Critical	8 (3.4)	3 (4.6)	5 (8.2)	0.410
Pneumonia	103 (81.8)	55 (84.6)	48 (78.7)	0.389
Symptoms at the admission, *n* (%)				
Dyspnoea	51 (40.4)	26 (40)	25 (41)	0.910
Cough	51 (40.4)	24 (36.9)	27 (44.3)	0.402
Asthenia	45 (35.7)	28 (43)	17 (27.9)	0.075
Anosmia/ageusia	6 (4.8)	4 (6.1)	2 (3.3)	0.449
Fever	83 (65.9)	46 (70.8)	37 (60.7)	0.231
Conjunctivitis	6 (4.8)	2 (3)	4 (6.6)	0.365
Asymptomatic	10 (7.9)	4 (6.1)	6 (9.8)	0.445
Respiratory features at the admission, median (IQR)				
SpO_2_	97 (95–98)	97 (95–98)	97 (95–98)	0.658
PO_2/_FiO_2_	367 (290–448)	362 (290–443)	400 (290–457)	0.375
FiO_2_	21 (21–21)	21 (21–21)	21 (21–21)	0.125
Laboratory findings at the admission, median (IQR)				
Haemoglobin, g/dl	11.7 (9.7–13.6)	11.4 (9.4–13.6)	12 (10.2–13.55)	0.948
White Blood Cells, × 10˄6/L	6380 (3790–10,660)	5630 (3140–9390)	7330 (4110–12,605)	0.175
Neutrophils, × 10˄6/L	4130 (2210–6820)	3650 (1950–7040)	4375 (2445–6765)	0.384
Lymphocytes, × 10˄6/L	900 (480–1630)	820 (410–1140)	1055 (505–2890)	**0.031**
Monocytes, × 10˄6/L	310 (160–490)	260 (120–410)	375 (175–570)	0.05
Platelets, × 10˄9/L	155 (105–207)	160 (113–202)	152 (103.5–215)	0.847
Thrombocytopenia (< 150 × 10˄9/L), *n* (%)	55 (43.6)	26 (40)	29 (48.3)	0.348
Neutropenia (< 500 × 10˄9/L), *n* (%)	12 (9.5)	9 (13.9)	3 (5)	0.093
Creatinine, mg/dl	0.9 (0.7–1.3)	0.9 (0.7–1.2)	0.95 (0.7–1.3)	0.653
Albumin, g/dl	36 (33–40)	36 (32–39)	37 (33–41)	0.323
D-dimer, µg/L	864 (478–1691.5)	831.5 (385–1551)	896 (623–1961)	0.163
C-Reactive Protein, mg/dL	4.5 (1.8–9.6)	3.46 (1.45–7.5)	4.8 (1.85–10.23)	0.209
Ferritin, µg/L	602.5 (210–1741)	935.5 (288–1743)	512 (140–1513)	0.186
LDH, U/L	296 (229–398)	290 (241–392)	311 (201–433)	0.971
Procalcitonin, ng/ml	0.24 (0.07–0.61)	0.24 (0.11–0.48)	0.24 (0.06–1.38)	0.999
Features at the respiratory worsening*				
Worsening during hospitalization, *n* (%)	65 (51.6)	38 (58.46)	27 (44.26)	0.111
FiO_2_ median (IQR)	35 (21–60)	35 (21–50)	30.5 (21–100)	0.508
PO_2/_FiO_2,_ median (IQR)	242 (146–350)	248 (163–374)	204 (89–324)	0.204
Days from SARS-CoV2 diagnosis to worsening, median (IQR)	8 (3–14)	7.5 (2–16)	6 (1–12)	0.412
Days from admission to worsening, median (IQR)	7 (2–17)	10 (3–17)	4 (1–12)	0.088
Late worsening (> 10 days), *n* (%)	88 (69.8)	47 (72.3)	41 (67.2)	0.533
Type of worse ventilation, *n* (%)				**0.046**
No oxygen	34 (27.0)	12 (18.5)	22 (36.1)	
Oxygen support by venturi mask	46 (35.5)	24 (36.9)	22 (36.1)	
HFNC/CPAP	32 (25.4)	18 (27.7)	14 (22.9)	
Mechanical ventilation	14 (11.1)	11 (16.2)	3 (4.9)	
Radiological findings at admission				
Lung involvement, %, median (IQR)	18.8 (5–40)	15 (5–35)	20 (5–50)	0.216
Classes of lung involvement, *n* (%) Low (0–30%) Medium (31–60%) High (> 60%)	87 (69.9) 25 (19.0) 14 (11.1)	48 (73.85) 11 (16.92) 6 (9.23)	39 (63.93) 14 (22.95) 8 (13.11)	0.484
Therapy				
Overall remdesivir therapy, *n* (%)	68 (53.9)	32 (49.2)	36 (59)	0.273
3 days remdesivir, *n* (%)	5 (3.9)	NA	5 (8.2)	**0.026**
5 days remdesivir, *n* (%)	63 (50)	32 (49.2)	31 (50.8)	0.900
Monoclonal antibodies, *n* (%)	37 (29.4)	NA	37 (63.8)	–
Remdesivir plus monoclonal antibodies, *n* (%)	23 (18.2)	NA	23 (62.2)	–
Oral antivirals, *n* (%)	1 (0.7)	NA	1 (1.64)	–
Tocilizumab, *n* (%)	9 (7.1)	6 (9.23)	3 (4.92)	0.348
Enoxaparin, *n* (%)	84 (66.6)	49 (75.4)	35 (57.3)	0.283
Corticosteroids, *n* (%)	98 (77.8)	59 (91)	39 (63.9)	**0.001**
Days of corticosteroids, median (IQR)	7 (2–17)	10.5 (8–20)	8 (6–10)	**0.002**
Baricitinib, *n* (%)	2 (1.6)	1 (1.6)	1 (1.64)	0.924
Convalescent plasma, *n* (%)	8 (6.3)	8 (12)	0 (0)	**0.004**
Antibiotics, *n* (%)	97 (77.0)	54 (83)	43 (70.5)	**0.04**
Vaccination, *n* (%)	55 (43.6)	NA	55 (90.2)	–
Number of vaccine doses, *n* (%)	3 (2–3)	NA	3 (2–3)	–
Last vaccine dose > 120 days, *n* (%)	22 (17.5)	NA	22 (40)	–
Outcomes				
Intensive care unit admission, *n* (%)	23 (18.3)	18 (27.7)	5 (8.2)	**0.005**
In-hospital mortality, *n* (%)	42 (33.3)	24 (36.9)	18 (29.51)	0.378
30-d mortality, *n* (%)	32 (25.4)	19 (29.2)	13 (21.3)	0.307
Days of viral shedding, median (IQR)	41 (32.5)	24 (15–50)	17 (10–28)	**0.011**
Secondary infections, *n* (%)	48 (38.1)	25 (38.5)	23 (37.7)	0.930
MDRO colonization, *n* (%)	14 (11.1)	9 (13.8)	5 (8.1)	0.399
Opportunistic infections, *n* (%)	14 (11.1)	8 (12.3)	6 (9.84)	0.659

Numbers in bold are statistically significant values (*p* < 0.05)

^*^Respiratory worsening was defined as: (i) the need of supplementary oxygen therapy or (ii) the need of increasing oxygen therapy supplementation in a patient with SARS-CoV2 infection for reasons directly related to the infection. A careful evaluation of causes of supplementary oxygen therapy for reasons other than SARS-CoV2 infection (i.e. cardiac failure, bacterial superinfections) was performed. In the case of doubt, a panel discussion was performed. MDRO: Multidrug resistant organisms. HFNC/CPAP: High-flow nasal cannula/continuous positive airway pressure

Non-Hodgkin lymphoma was the most frequent underlying HM in both groups (40% vs. 44.3%, *p* = 0.628) followed by chronic lymphocytic leukaemia (10.8% vs. 14.7%, *p* = 0.598) and multiple myeloma (15.4% vs. 6.6%, *p* = 0.115). No difference was observed between the two groups when considering active malignancy rates (41.5% vs. 50.8% in PRE- and POST-V-mAb groups, respectively, *p* = 0.223); in detail, patients in the PRE-V-mAb group were less likely to have a relapsing or refractory disease (18.5% vs. 34.4%, *p* = 0.042) than patients in the POST-V-mAb group and more likely to have a complete or partial remission of underlying HM (53.8% vs. 34.4%, *p* = 0.028). A similar proportion of patients in both groups received prior active treatment (55.4% vs. 52.5%, *p* = 0.742) (Supplementary Table 1).

The use of corticosteroids (91.0% vs. 63.9%, *p* = 0.001) and antibiotics (83.0% vs. 70.5%, *p* = 0.04) was significantly higher in the PRE-V-mAb group, while no difference was observed in treatment with remdesivir (49.2% vs. 50.8%, *p* = 0.861), enoxaparin (75.4% vs. 57.3%, *p* = 0.283), tocilizumab (9.2% vs. 4.9%, *p* = 0.348) and baricitinib (1.6% vs. 1.6%, *p* = 0.924) (Table 1).

In the POST-V-mAb group, 37 (63.8%) patients received mAb, 31 (50.8%) received remdesivir and 23 (62.2%) received remdesivir plus mAbs. The majority of patients (55, 90.5%) had been previously vaccinated against SARS-CoV2, 22 of whom (40.0%) have received the last vaccine dose more than 120 days before hospital admission. The median number of vaccine doses was 3 (2–3).

### Clinical outcomes and risk of in-hospital mortality

Patients in the PRE-V-mAb group showed a significantly higher risk of ICU admission (27.7% vs. 8.2%, *p* = 0.005) and longer hospital length (20 vs. 13 days, *p* = 0.0003) than patients in the POST-V-mAb group. Conversely, both in-hospital and 30-day mortality (36.9% vs. 29.5% and 29.2% vs. 21.3% in PRE- and POST-V-mAb groups, *p* = 0.378, *p* = 0.307, respectively) rates did not significantly differ between the two groups, even though they tend to be lower in the POST-V-mAb group.

At multivariable analysis, an active malignancy (Hazard ratio—HR -2.20; 95% confidence interval—CI—1.02–4.72; *p* = 0.042), a critical COVID-19 at admission (HR 2.99; 95% CI, 1.15–7.80; *p* = 0.025) and the need for high level of oxygen support at respiratory worsening [either high-flow nasal cannula/continuous positive airway pressure, HFNC/CPAP (HR 5.96; 95% CI, 1.29–27.52; *p* = 0.022) or mechanical ventilation (HR 7.63; 95% CI, 1.59–36.62; *p* = 0.011)] were independently associated with an increased risk of in-hospital mortality in the overall population (Table 2).

**Table 2:** Analysis of risk factors for in-hospital mortality in patients with haematological malignancies

Cox regression model	HRs (CIs 95%)	*p*-value
Sex (female)	0.67 (0.31–1.44)	0.307
Age > 65y	2.02 (0.86–4.72)	0.103
Severity of infection (critical vs non-critical)	2.99 (1.15–7.80)	**0.025**
Type of haematological malignancy (compared to ALL)		
AML	2.21 (0.23–21.00)	0.490
CLL	0.98 (0.10–9.83)	0.989
MM	1.01 (0.10–9.60)	0.992
NHL	0.94 (0.11–7.72)	0.959
HL	1.37 (0.06–27.08)	0.834
Myelodysplastic syndrome	0.51 (0.28–9.47)	0.658
Myelofibrosis	1.37 (0.07–27.32)	0.835
Active malignancy°	2.20 (1.02–4.72)	**0.042**
Group of radiological involvement, % (compared to minimum, 0–30%)		
Medium (31–60%)	0.85 (0.35–2.06)	0.734
High (> 60%)	1.29 (0.48–3.45)	0.611
Type of worse oxygen support at respiratory worsening* (compared to no oxygen support)		
Oxygen	1.11 (0.21–5.83)	0.898
HFNC/CPAP	5.96 (1.29–27.52)	**0.022**
MV	7.63 (1.59–36.62)	**0.011**

Numbers in bold are statistically significant values (*p* < 0.05)

°Active malignancy includes patients with new diagnosis or relapsing/refractory disease; *: respiratory worsening was defined as: (i) the need of supplementary oxygen therapy or (ii) the need of increasing oxygen therapy supplementation in a patient with SARS-CoV2 infection for reasons directly related to the infection. A careful evaluation of causes of supplementary oxygen therapy for reasons other than SARS-CoV2 infection (i.e. cardiac failure, bacterial superinfections) was performed. In the case of doubt, a panel discussion was performed. ALL: Acute lymphocytic leukaemia; AML: Acute myeloid leukaemia; CLL: Chronic lymphocytic leukaemia; MM: multiple myeloma; NHL: Non-Hodgkin lymphoma; HL: Hodgkin lymphoma; HFNC: High-flow nasal cannula; CPAP: continuous positive airway pressure; MV: mechanical ventilation

When just the POST-V-mAb group was included in the model, mAbs administration (HR 0.03; 95% CI, 0.002–0.76; *p* = 0.033) was independently associated with a reduced risk of in-hospital mortality, whereas the severity of infection at admission (HR 5.75; 95% CI 1.01–32.74; *p* = 0.048) confirmed its association with a worse outcome (Supplementary Table 2).

### Duration and risk of prolonged viral shedding (PVS)

Overall, median duration of viral shedding was 20.5 days (IQR, 18.7–22.2) and was higher in PRE-V-mAb than in POST-V-mAb group [24 (IQR, 15–50) vs. 17 (IQR, 10–28) days; *p* = 0.011]. Baseline characteristics of patients stratified according to viral shedding (i.e. < 20 vs. > 20 days) are reported in Table 3.

**Table 3:** Features of study population according to short (≤ 20 days) or prolonged (> 21 days) viral shedding

	Short VS (*N* = 62)	Long VS (*N* = 64)	*p* value
Demographics			
Gender (Female), *n* (%)	24 (38.7)	27 (42.2)	0.691
Age, median (IQR)	68.5 (57–79)	68.5 (54–76)	0.705
Steroids in the last 30 days before admission, *n* (%)	19 (31.2)	24 (37.5)	0.455
Infection in the last 30 days before admission, *n* (%)	9 (14.5)	14 (21.9)	0.285
Antibiotic therapy in the last 30 days (excluded prophylaxis), *n* (%)	17 (27.4)	22 (34.4)	0.398
Days of hospitalization, median (IQR)	10 (7–16)	35.5 (16.5–50)	**0.0001**
Days from symptoms onset to hospitalization, median (IQR)	2 (1–5)	3 (1–7)	0.206
Active treatment (previous 90-d)	27 (43.6)	41 (64.1)	**0.021**
Active treatment: chemotherapy only	18 (29.0)	22 (34.38)	0.520
Active treatment: immunotherapy only	4 (6.45)	17 (26.56)	**0.002**
Active treatment: chemotherapy plus immunotherapy	6 (9.7)	9 (14.1)	0.346
Chemotherapy (previous 30-d)	18 (29.5)	17 (25.6)	0.714
Comorbidities, *n* (%)			
Hypertension	30 (48.4)	22 (34.4)	0.110
Coronary artery disease	16 (25.8)	12 (18.8)	0.341
Diabetes	7 (11.3)	910 (15.6)	0.476
Chronic obstructive pulmonary disease	4 (6.5)	4 (6.3)	0.963
Chronic renal failure	6 (9.7)	3 (4.7)	0.277
Disease severity at the admission, *n* (%)			
Moderate	43 (69.4)	37 (57.8)	0.179
Severe	14 (22.6)	17 (26.6)	0.604
Critical	4 (6.5)	4 (6.3)	0.963
Pneumonia	51 (82.3)	52 (81.3)	0.884
Features at the respiratory worsening*			
Worsening during hospitalization, *n* (%)	29 (46.8)	36 (56.3)	0.287
Days from SARS-CoV2 diagnosis to worsening, median (IQR)	3.5 (1–9)	13 (4–30)	**0.0005**
Days from admission to worsening, median (IQR)	3 (1–7)	14.5 (3–24.5)	**0.0003**
Late worsening (> 10 days), *n* (%)	38 (61.3)	50 (78.1)	**0.040**
Therapy			
Overall remdesivir therapy, *n* (%)	40 (64.5)	27 (43.6)	**0.019**
Duration of remdesivir therapy, days (median, IQR)	5 (5–5)	5 (5–5)	0.421
Monoclonal antibodies, *n* (%)	21 (34.4)	16 (25.8)	0.297
Remdesivir plus monoclonal antibodies, *n* (%)	18 (45.0)	5 (17.2)	**0.016**
Oral antivirals, *n* (%)	0 (0)	1 (1.5)	–
Tocilizumab, *n* (%)	3 (4.8)	6 (9.4)	0.323
Corticosteroids, *n* (%)	42 (72.4)	56 (93.3)	**0.002**
Days of corticosteroids, median (IQR)	8 (6–11)	10 (8–22)	**0.001**
Duration of corticosteroids > 7 days	44 (72.1)	55 (85.9)	0.057
Baricitinib, *n* (%)	0 (0)	2 (3.2)	0.154
Convalescent plasma, *n* (%)	0 (0)	8 (12.7)	**0.004**
Antibiotics, *n* (%)	45 (72.6)	52 (83.9)	0.128
Vaccination, *n* (%)	33 (53.2)	22 (34.4)	0.033
Last vaccine dose > 120 days, *n* (%)	18 (36.0)	26 (46.4)	0.277
Outcomes			
Intensive care unit admission, *n* (%)	11 (17.7)	12 (18.8)	0.884
In-hospital mortality, *n* (%)	22 (35.5)	20 (31.3)	0.614
Secondary infections, *n* (%)	20 (32.3)	28 (43.7)	0.184

Numbers in bold are statistically significant values (*p* < 0.05)

^*^Respiratory worsening was defined as: (i) the need of supplementary oxygen therapy or (ii) the need of increasing oxygen therapy supplementation in a patient with SARS-CoV2 infection for reasons directly related to the infection. A careful evaluation of causes of supplementary oxygen therapy for reasons other than SARS-CoV2 infection (i.e. cardiac failure, bacterial superinfections) was performed. In the case of doubt, a panel discussion was performed. VS: viral shedding

According to the logistic regression model shown in Table 4, active treatment for the HM in the previous 90 days (OR 3.55; 95% CI, 1.35–9.32; *p* = 0.010), respiratory worsening after more than 10 days (OR, 2.92; 95% CI, 1.05–8.05; *p* = 0.038), use of corticosteroids (OR, 7.59; 95% CI, 1.73–33.24; *p* = 0.007) and its duration for > 7 days (OR, 4.92; 95% CI, 1.59–15.22; *p* = 0.006) were risk factors independently associated with a prolonged viral shedding.

**Table 4:** Analysis of risk factors for prolonged (> 20 days) viral shedding

Logistic regression model°	ORs (CIs 95%)	*p*-value
Active treatment (previous 90-d)	3.55 (1.35–9.32)	**0.010**
SARS-CoV-2 vaccination	0.94 (0.26–3.33)	0.926
Corticosteroids	7.59 (1.73–33.24)	**0.007**
Respiratory worsening* > 10 days	2.92 (1.05–8.05)	**0.038**
Duration of corticosteroid therapy > 7 days	4.92 (1.59–15.22)	**0.006**
Type of therapy for SARS CoV2 infection (compared to no therapy) RDV only Monoclonal antibodies only RDV + monoclonal antibodies	0.30 (0.09–0.955) 2.09 (0.31–13.81) 0.16 (0.02–1.07)	**0.042** 0.444 0.060

Numbers in bold are statistically significant values (*p *< 0.05)

°Model adjusted for age and sex. *: respiratory worsening was defined as: (i) the need of supplementary oxygen therapy or (ii) the need of increasing oxygen therapy supplementation in a patient with SARS-CoV2 infection for reasons directly related to the infection. A careful evaluation of causes of supplementary oxygen therapy for reasons other than SARS-CoV2 infection (i.e. cardiac failure, bacterial superinfections) was performed. In the case of doubt, a panel discussion was performed. RDV: remdesivir

## Discussion

In the present study, we observed that hospitalized patients with HM and COVID-19 continued to present high mortality rates despite the introduction of vaccination and mAbs. However, we could show a significant reduction in terms of ICU admission, hospitalization length and duration of viral shedding compared with the first pandemic waves. Notably, our results highlight how both the severity of COVID-19 at admission and at respiratory worsening and the presence of an active malignancy at the time of SARS-CoV2 diagnosis represent the major drivers of mortality.

In the literature, the mortality rate reported on hospitalized patients widely varied, ranging from 12 to 23%, but still significantly lower than the previous periods [6, 27, 28]. More recently, results from the international platform EPICOVIDHEA [29] reported mortality rates of 11.5% and 16% in a vaccinated hospitalized population affected by HM in 2021 and patients hospitalized due to Omicron infection, respectively [27, 28]. On the other hand, preliminary data from a Danish study focussing only on Omicron-infected chronic lymphocytic leukaemia patients and a Japanese case series observed a 23% and 22% 30-day mortality rate, respectively [30, 31].

In our study, the mortality rates were higher, although a slight, but not significant, reduction was observed after vaccination and mAb. However, given the rapid pandemic evolution and the succeeding of new variants, which may overlap during the same period of time and differ in circulation among countries, consistent and homogeneous data on outcomes in patients with HM and the effects of vaccination, mAbs and oral antivirals are still lacking. Furthermore, recent studies focussed on both non-hospitalized and hospitalized patients or only on a particular type of HM or a specific variant, circumstances that may by definition influence the outcome. Consequently, it is very difficult to make a direct and crude comparison among different studies.

We found that the independent predictors of mortality were the severity of infection at admission and at respiratory worsening and the presence of an active malignancy, prompting that COVID-19 and HM might have a co-attributable role in the mortality of hospitalized patients with HM despite advance in SARS-CoV-2 prevention and treatment.

These findings indeed highlight how the COVID-19 still represent *itself* a major challenge in HM patients, since, although reduced after the implementation of vaccination and mAb, a nonnegligible rate of subjects experienced a respiratory worsening needing oxygen support exclusively as a consequence of infection’s progression. At the same time, an active malignancy at the time of SARS-CoV2 diagnosis may concur to a worse outcome [32]. Indeed, in our cohort of patients more than half of subjects had an active malignancy. When looking in depth, there was a statistically significant higher percentage of patients with relapsing or refractory disease in the POST-V-mAb group, which may have contributed to the observed high mortality in this setting.

Furthermore, patients in the POST-V-mAb group were admitted at a time when first the Delta and only later the Omicron variants were predominant in Italy. Even though Omicron variant has a higher transmission rate but less severity than previous ones, the presence of Delta variant could also explain our higher fatality rate compared with last published studies strictly under conditions of Omicron dominance.

In the subgroup of patients in the POST-V-mAb, we could observe that receiving mAb was a protective factors for mortality, suggesting the advantage of early therapies in this high-risk group of patients and in line with a recent published paper by the EPICOVIDHEA group [33]. Due to the few patients who received oral antivirals, we could not generalize this suggestion on the use of oral antivirals and additional studies are warranted.

In line with the literature [18, 34–37], we found a reduction on ICU admission rates. These findings may be explained with the lower need of high-level oxygen support (i.e. CPAP/HFNC and MV) observed in the POST-V-mAb group.

Another interesting finding was the analysis of the duration of viral shedding in this high-risk population. First, we confirmed that HM patients exhibit a prolonged shedding of viral RNA from the upper respiratory tract [8], which has a crucial impact on the decision whether to start or continue the treatment of the HM, especially in patients with active malignancy. Nevertheless, the observed reduction of viral shedding observed in the POST-V-mAb group has important consequences: on the one hand, it increases the opportunity for the patients to receive the treatment of underlying disease; on the other hand, it may reduce the possibility of selecting viral variants, which may be less susceptible to patients’ immunity and to drugs’ activity [38, 39]. Second, we assessed the predictors of PVS and found that not only corticosteroid use (either for the treatment of the underlying disease or COVID-19) but also its duration for more than 7 days was independently associated with PVS [40, 41].

A recent study highlighted that not only clinical outcomes, but also viral clearance was positively influenced by the association with remdesivir/dexamethasone [42]. The use of remdesivir may therefore counterbalance the effect of corticosteroids towards PVS and may suggest that an early and timely use of antivirals should be strongly recommended, especially in immunosuppressed patients with HM.

This study undoubtedly presents several limitations. Firstly, it is a retrospective and single-centre nature; furthermore, the small study population did not allow us to stratify our patients by the different types of HM, and due to few patients who received oral antivirals, we could not assess the efficacy of this treatment in HM population. Secondly, we were unable to identify the SARS-CoV-2 variant of concern in all infected patients; therefore, the variant was indirectly defined based on the dominant one in Italy in the same period. However, the aim of the study was not to investigate the effect of a specific variant on the outcome, which, in our opinion, deserves targeted studies. Unfortunately, we were not able to recollect data of the exact timing of mAbs administration in the outpatient setting. Finally, it is quite complicated to speculate whether the treatment of the underlying HM would have affected mortality in those who were not treated. In addition, there was a lack of data on the efficacy of oral antivirals and the long-acting mAbs, which need further targeted studies in the haematological population.

In conclusion, we showed that vaccination and mAbs reduce ICU admission, hospitalization length and viral shedding in COVID-19 patients with HM, who, however, still remain a high-vulnerable population with high mortality rates. Strict adherence to non-pharmacological interventions and vaccinations remains mandatory for this frail population in the time where SARS-CoV-2 is continuously circulating in the community.

## Supplementary Information

Below is the link to the electronic supplementary material.

Supplementary file1 (DOCX 27 KB)
