# Supplementary material for: Outcome of COVID-19 patients with haematological malignancies after the introduction of vaccination and monoclonal antibodies: results from the HM-COV 2.0 study
Source: Clin Exp Med. 2023 Mar 3;23(6):2275–85. doi: 10.1007/s10238-023-01027-y (PMC9982764; doi:10.1007/s10238-023-01027-y)
Supplement: Supplementary file 1 — Supplementary file1 (DOCX 27 KB) [file 10238_2023_1027_MOESM1_ESM.docx]

**Supplementary data**

**This file presents:**

- **Supplementary Table 1, p 1;**
- **Supplementary Table 2, p 2;**

|  | **PRE-V-mAb**  **(N=65)** | **POST-V-mAb**  **(N=61)** | ***p* value** |
| --- | --- | --- | --- |
| **Disease status at the diagnosis of SARS-CoV2 infection°** |  |  |  |
| New diagnosis | 15 (23.1) | 10 (16.4) | 0.462 |
| Complete or partial remission | 35 (53.8) | 21 (34.4) | **0.028** |
| Relapsing or refractory disease | 12 (18.5) | 21 (34.4) | **0.042** |
| Not defined disease status | 3 (4.62) | 9 (14.75) | 0.113 |
| Active malignancy°° | 27 (41.5) | 31 (50.8) | 0.223 |
| **Treatment of the underlying malignancy** |  |  |  |
| Active treatment in the last 90 days* | 36 (55.4) | 32 (52.5) | 0.742 |
| Chemotherapy | 20 (30.8) | 20 (32.8) | 0.808 |
| Immunotherapy | 11 (16.9) | 10 (16.4) | 0.936 |
| Chemotherapy plus immunotherapy | 9 (13.9) | 5 (8.2) | 0.363 |
| Chemotherapy in the last 30 days | 21 (32.3) | 14 (22.9) | 0.264 |
| Prior/concluded treatment | 28 (43.1) | 34 (55.7) | 0.085 |
| Allogeneic stem cell transplantation | 4 (6.1) | 2 (3.2) | 0.799 |
| **Type of haematological disease** |  |  |  |
| Acute lymphocytic leukemia | 5 (7.7) | 3 (4.9) | 0.523 |
| Acute myeloid leukemia | 7 (10.8) | 2 (3.2) | 0.165 |
| Chronic myeloid leukemia | 0 (0) | 3 (4.9) | 0.070 |
| Chronic lymphocytic leukemia | 7 (10.8) | 9 (14.7) | 0.598 |
| Plasma cell leukemia | 1 (1.5) | 0 (0) | 0.331 |
| Multiple myeloma | 10 (15.4) | 4 (6.6) | 0.115 |
| Essential thrombocythemia | 1 (1.5) | 0 (0) | 0.331 |
| Myelodysplastic syndrome | 1 (1.5) | 3 (4.9) | 0.353 |
| Myelofibrosis | 3 (4.6) | 2 (3.3) | 0.701 |
| Non-Hodgkin lymphoma | 26 (40) | 27 (44.3) | 0.628 |
| Hodgkin lymphoma | 1 (1.5) | 3 (4.9) | 0.353 |
| Polycythemia vera | 1 (1.5) | 1 (1.6) | 0.964 |

**Supplementary Table 1.** Disease features of patients with haematological malignancy.

°: Status of haematological malignancy was defined as new diagnosis, refractory or relapsing disease, according to the guidelines of European Society for Medical Oncology [24].

°°: Active malignancy includes patients with new diagnosis or relapsing/refractory disease;

*: Prior active treatment included the receipt of chemotherapy or immunotherapy, or both, in the previous 90 days.

Immunotherapy included the receipt of monoclonal antibodies as rituximab, daratumumab, obinutuzumab and tyrosine kinase inhibitors as imitinib, ibrutinib, ruxolitinib, venetoclax. Numbers in **bold** are statistically significant values (p< 0.05).

**Supplementary Table 2.** Analysis of risk factors for in-hospital mortality in the POST-V-mAb group of patients.

| Cox regression model^§^ | HRs (CIs 95%) | *p-value* |
| --- | --- | --- |
| Severity of infection (critical *vs* non-critical) | 5.75 (1.01-32.74) | **0.048** |
| Active malignancy° | 1.82 (0.52-6.33) | 0.344 |
| Type of therapy for SARS CoV2 infection (compared to no therapy)  RDV only  Monoclonal antibodies only  RDV+Monoclonal antibodies only | 0.56 (0.06-5.51)  0.03 (0.002-0.76)  0.15 (0.01-2.00) | 0.621  **0.033**  0.152 |

§: model adjusted for type of haematological malignancies.

°: Active malignancy includes patients with new diagnosis or relapsing/refractory disease.

RDV: remdesivir. Numbers in **bold** are statistically significant values (p< 0.05).
